# The benefits of mindfulness in mental healthcare professionals

**DOI:** 10.12688/f1000research.73729.1

**Published:** 2021-10-25

**Authors:** Tayler Watson, Owen Walker, Robin Cann, Ashwin K Varghese

**Affiliations:** 1 Mental Health, Drugs and Alcohol Service, Barwon Health, Geelong, Victoria, 3220, Australia; 2 Department of Psychiatry, Monash Health, Melbourne, Victoria, 3806, Australia; 3 Nova Scotia Health Authority, Halifax, Nova Scotia, NS B3S 0H6, Canada

**Keywords:** Mindfulness, Mental Health, Burnout, Psychiatry

## Abstract

**Background:** Burnout is a widely reported syndrome consisting of emotional exhaustion, depersonalization, and a lowered sense of accomplishment. Mindfulness practices have been shown to be useful in lowering distress and burnout in clinical and non-clinical cohorts. Our aim was to explore the potential personal and occupational benefits of a structured mindfulness intervention on a cohort of mental health professionals. A mixed-methods approach was utilised in order to enhance the exploratory power of the study. **Methods**: We conducted a pilot study involving healthcare practitioners employed at a community outpatient mental health clinic. As a pilot, we relied on a single group and implemented a quasi-experimental, simultaneous mixed methods design by incorporating both quantitative pre- and post- testing alongside written qualitative post-test responses. **Results**: Analysis of the data demonstrated a significant difference between overall mindfulness when comparing post-test (mean=140.8, standard deviation=18.9) with pre-test data (mean=128.3, standard deviation=28.6). Participants also showed a statistically significant difference in three of the subscales: observation, describing, and non-reactivity. A moderate effect size was seen for each of the above differences.  Analysis of the qualitative data revealed a range of potential themes which may be used to explain the differences exhibited across participants’ personal and professional lives, which can be grouped into two thematic overarching groups: emotional reactivity and listening/communicating. **Conclusions**: The results of this pilot study indicate that a structured, six-week mindfulness program has the potential to benefit clinicians, personally by reducing emotional reactivity and professionally by promoting deep listening and communication.

## Introduction

Proposed by Maslach *et al*. in 1986, burnout is now widely accepted as consisting of emotional exhaustion, depersonalization and a lowered sense of accomplishment, contributing to an overall reduction in psychological wellbeing. ^1^ High levels of burnout have been recorded across industries ^2^^–^^4^ with common etiological themes related to workload demands, low occupational satisfaction and psychosocial stressors. ^5^^–^^7^ Some reports suggest up to 80% of physicians meet criteria for burnout, ^8^ however more conservative estimates suggest around half of physician’s experience burnout. ^9^ Up to 78% of psychiatrists and 21% of psychiatry residents are experiencing burnout at any one time. ^10^^,^^11^ Around half of family physicians report high levels of emotional exhaustion and depersonalization, and more than two thirds of young oncologists report burnout. ^12^^,^^13^ Two thirds of mental health clinicians including psychologists, social workers and nurses, also report distress. ^14^ Burnout is associated with major depression, poor patient outcomes and more negative feelings towards patients. ^15^^–^^17^ Physician burnout also results in significant economic loss. ^18^ By contrast, psychiatry is well placed to drive positive change. ^19^ It is clear that burnout is a key issue facing the medical and psychosocial professions. While the existence of the phenomenon is well understood, attempts to rectify the problem remain scarce. Interventions would need to be easily administered and suitably scalable. Mindfulness represents a promising avenue for exploration, given the ease with which it can be taught and maintained.

Mindfulness can be defined as “paying attention to present moment experiences with openness and curiosity and a willingness to be with what is” ( UCLA health). Operational definitions distinguish two components of mindfulness: self-regulation of attention and orientation to the present moment characterized by curiosity, openness and acceptance. ^20^ A range of mindfulness practices and techniques have been developed and applied for use in both mental and physical illnesses. ^21^ One of these practices, Mindfulness Based Stress Reduction (MBSR), demonstrates efficacy in reducing stress, depression, anxiety and negative ruminations. ^22^^–^^24^ MBSR delivered to therapists in training enhanced self-compassion and positive affect. ^25^ When applied to physicians, MBSR may reduce burnout, enhance resilience and improve patient care. ^26^^–^^28^ Mindfulness has also been correlated with neurophysiological and neurobiological changes in key brain regions associated with the observed phenomenology. ^29^

This preliminary pilot study aims to explore the benefits of a mindfulness intervention on mental health practitioners. Mixed methods approaches have been shown to be an effective way to allow for a more robust exploration of a phenomenon than each individual method used in isolation. ^30^ Collecting both types of data can greatly assist in the exploratory nature of the study, with qualitative information used to further explore, as well as potentially explain, the quantitative analysis.

## Methods

### Study design

This pilot study employed a single group quasi-experimental, simultaneous mixed-methods design by utilizing quantitative pre and post testing paired with a written qualitative post-assessment. The qualitative component of this study used a descriptive phenomenological method.

### Participants and recruitment

13 healthcare practitioners employed at a community outpatient mental health clinic in Amherst, Nova Scotia were recruited for the study in October 2016 ( Table 1). Recruitment was completed via email and word-of-mouth discussion. Interested clinicians were then asked to refer colleagues who were not reached via email. Non-probability convenience sampling was used to recruit participants with desired study characteristics and expeditiously collect qualitative data. Snowball sampling increased the potential number of participants. This number of participants was chosen as evidence suggests theoretical saturation occurs at 12 respondents. ^31^^,^^32^

**Table 1.** Displays the total number and percentage of participants (n = 13) by role and gender.

**Clinical role**	Psychiatrists	6 (46%)
	Social Workers	6 (46%)
	Family Physician	1 (7%)
**Gender**	Male	4 (31%)
	Female	9 (69%)

### Mindfulness program

The Mindfulness Awareness Practices (MAPs) program is a six-week, validated protocol delivered by a trained instructor. The intervention has demonstrated usefulness in reducing distress in clinical populations. ^33^^,^^34^ Participants completed the MAPs six-week course, in November and December 2016. Week one included a two-hour workshop focussed on a conceptual introduction to mindfulness and psychoeducation. In weeks two to five, participants engaged in weekly didactic and experiential 90-minute workshops learning a range of mindfulness techniques, such as sitting, relational and movement meditation and skills for recognising positive and difficult emotions. Participants were encouraged to complete and record ‘homework’ activities, including guided meditation practice. The program was delivered on-site at an outpatient community mental health clinic during the participants’ regular workday.

### Questionnaires

The Five Facet Mindfulness Questionnaire (FFMQ), first proposed in 2006, is a robust, 39-item self-report, Likert type scale measuring five correlates of mindfulness; observing, describing, acting with awareness, non-judging and non-reacting. ^35^^,^^36^ The survey’s factor structure is available in the original article. ^35^ The scale exhibits reliable construct validity across facets. ^37^ Examples of statements include “I’m good at finding words for my feelings” and “I watch my feelings without getting lost in them”. Respondents are then asked to rate these statements with five potential options (never or very rarely true, rarely true, sometimes true, often true, very often or always true).

The Perceived Stress Scale (PSS) ^10^ is an extensively used self-report questionnaire designed to measure individual stress, demonstrating high internal consistency across populations ( α= 0.82). ^38^^,^^39^ Respondents are asked 10 questions, scored on a Likert scale (never, almost never, sometimes, fairly often or very often). Questions assess the level of perceived stress one has experienced within the last month. For example,”in the last month, how often have you been upset because of something that happened unexpectedly”? The scale is widely available and has been validated in several languages. ^40^^–^^42^

Participants completed handwritten, hardcopy FFMQ and PSS questionnaires prior to the first session (week 1) and post-participation in the protocol (week 6). Qualitative data was obtained using brief, open-ended written survey questions that allowed for narrative-style feedback from participants, completed post-intervention. The questions were developed to allow for rich and open responses in the context of the study design and intervention, and therefore were not based on pre-existing models or surveys. The questions were as follows:

“In what way has the six-week mindfulness training influenced your personal life?”

“In what way has the six-week mindfulness training influenced your clinical practice?”

### Statistical analysis

FFMQ and PSS scores were set as primary outcome measures. Continuous data from the quantitative questionnaires was entered into SPSS (version 24). Paired samples t-tests were then conducted. Statistical significance was assumed at 0.05%. Corresponding *Cohen's d* values were determined, and effect sizes established. Outcome variables included overall mindfulness, perceived stress and the five FFMQ subscales. Secondary outcome measures sought to explore the relationship between engagement and change in outcome scores, however statistical analysis was impacted by poor response rates.

### Data analysis

Participants were provided with the two questions stated above, and in turn provided the researchers with written responses. Written responses were transcribed to text using Microsoft Word (version 16.53) and some minor substitutions (e.g., replacing shorthand with full words) were made to aid legibility, with no impact to the meaning of the responses. 1% of words were illegible, despite consulting a second reviewer. Thematic analysis was applied to the qualitative data to observe themes that arose across participants’ varying experiences of the program. This allowed the authors to identify, analyse and describe recurrent themes and concepts observed within the dataset. Thematic analysis is a flexible approach that provides a framework for a rich discussion. ^43^ Considering the subjectivity of the research topic, the authors adopted this approach to best reflect the true experience of the participants, and thus enhance trustworthiness.

### Reflexive statement

The researchers acknowledge their own characteristics in the interpretation of the qualitative data. Interpreting authors were predominantly male from medical backgrounds with special interests in the subject matter, which we recognise may have impacted objectivity.

### Ethics and consent

The Nova Scotia Health Authority Research Ethics Board granted an exemption from ethics approval as the study is a quality improvement/educational initiative. Verbal, informed consent was obtained from participants prior to commencement for use and publication of study data as all participants were staff of the health authority. The paper forms were stored in a locked cabinet and digital data were stored on password-protected computers.

## Results

### Primary outcome analysis

**Figure 1.**
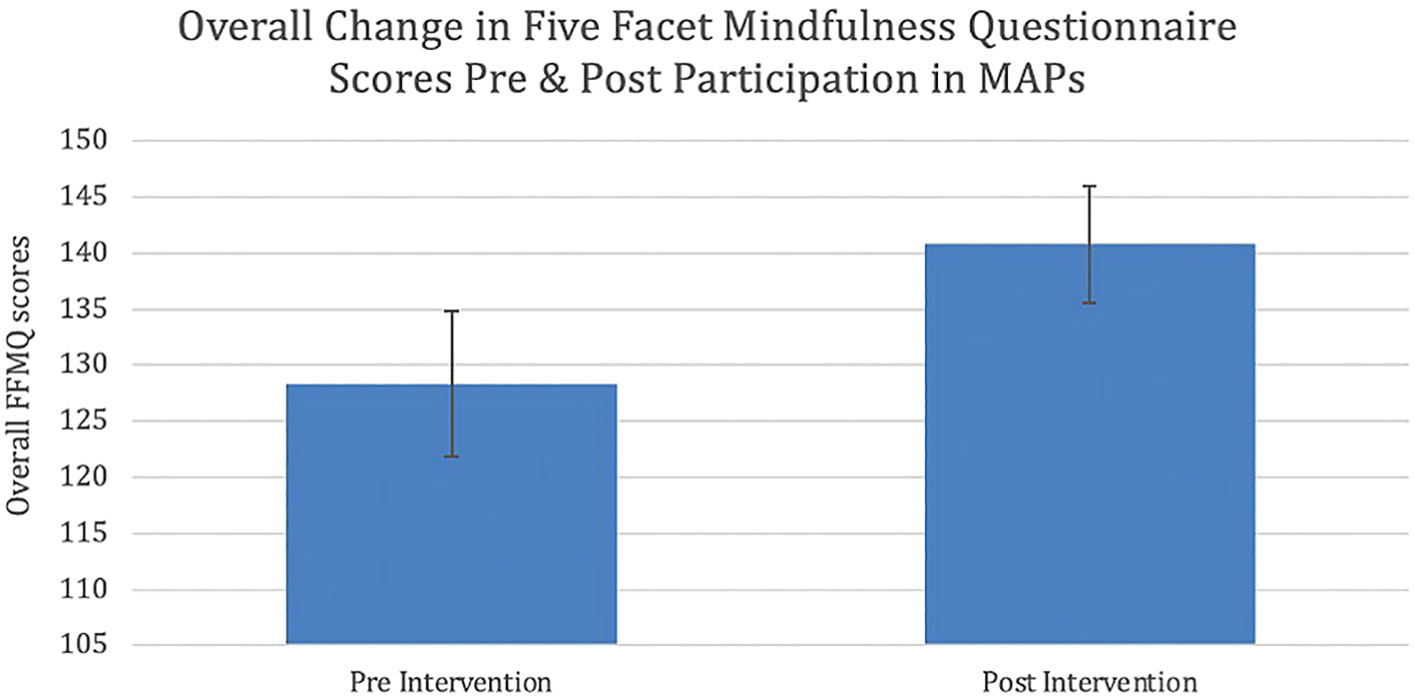
Mean change in overall FFMQ scores pre and post participation in MAPs protocol (p = 0.007). Error bars represent standard errors (SEM). FFMQ: Five Facet Mindfulness Questionnaire. MAPs: Mindfulness Awareness Practices.

**Figure 2.**
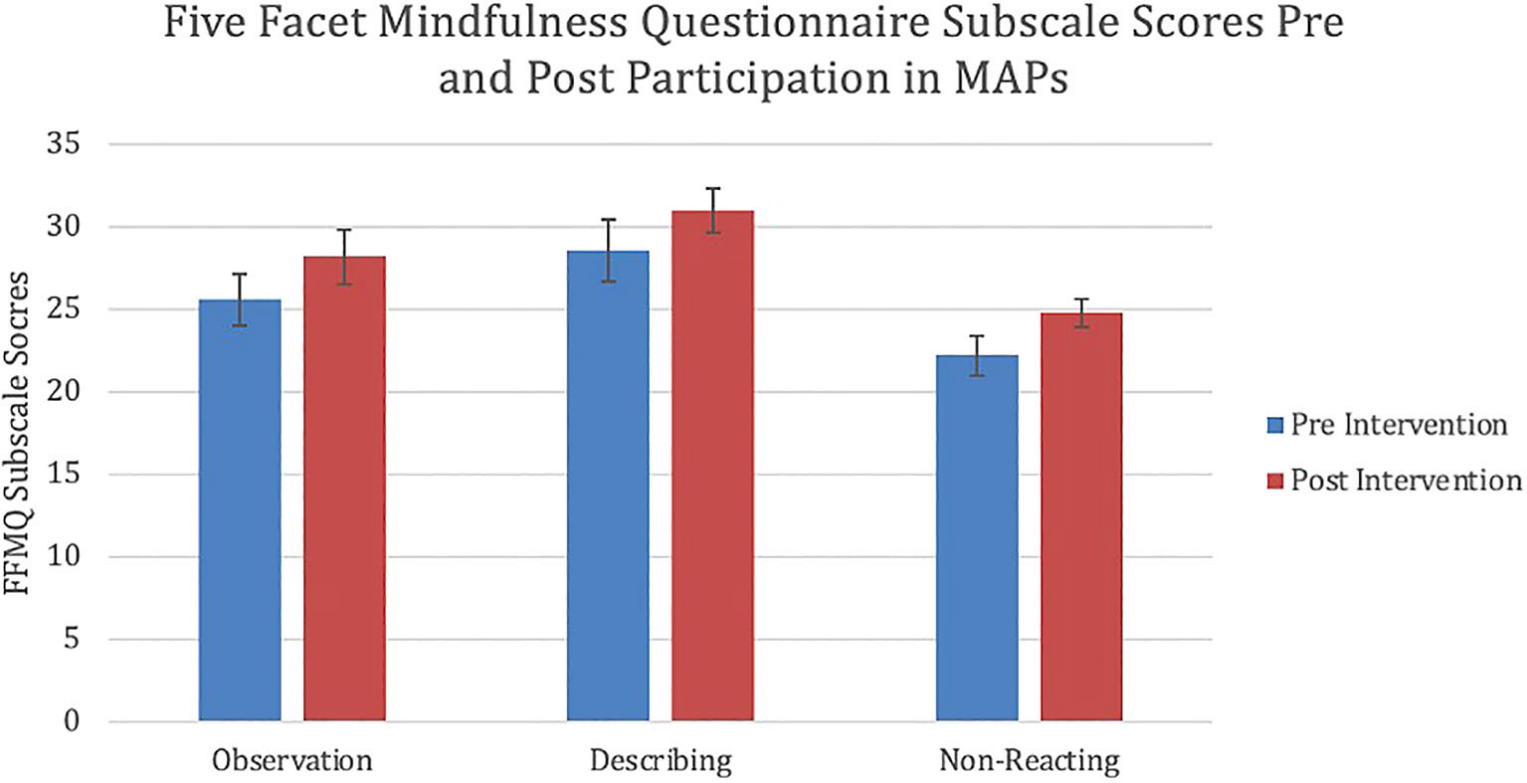
Mean change in observation (p = 0.036), describing (p = 0.042) and non-reacting (p = 0.019) FFMQ subscale scores following participation in MAPs. Error bars represent standard errors (SEM). FFMQ: Five Facet Mindfulness Questionnaire. MAPs: Mindfulness Awareness Practices.

Demographic characteristics of the cohort are displayed in Table 1. Most participants were female and either worked in psychiatry or social work. Table 2 displays statistically significant results of paired sample T-tests, used to measure pre-post change in overall mindfulness and FFMQ subscales. On the FFMQ, there was a statistically significant difference in overall mindfulness after participation in MAPs when compared to before participation (p = 0.007) ( Figure 1). Participants also demonstrated statistically significant changes on three subscales of the FFMQ: observation, describing and non-reactivity ( Figure 2). There was a statistically significant increase in participants’ ability to observe internal and external experiences when compared to baseline (p = 0.036). Similarly, a statistically significant increase was also demonstrated in participants’ ability to describe and label internal experiences after participation in MAPs than at the time of pre-test (p = 0.042). Finally, participants showed reduced reactivity to internal experiences and enhanced ability to allow the free flow of thought without rejection after participation in the study when compared to baseline (p = 0.019). Moderate effect sizes were demonstrated in the overall FFMQ (Cohen’s d = 0.588), observing (Cohen’s d = 0.452), describing (Cohen’s d = 0.407) and non-reacting (Cohen’s d = 0.693) subscales. The mean number of absences to sessions was 1.2. Reason for absences was not recorded. Three participants missed two or more sessions in total. No statistically significant difference in FFMQ scores was observed in this group (p = 0.98). There were no statistically significant changes on the final two subscales of the FFMQ, awareness and non-judging; as well, no statistically significant changes in the perceived stress scale were observed.

**Table 2.** Displays mean change of FFMQ scales pre and post participation in MAPs, displayed with corresponding t and p values. Corresponding effect sizes presented as Cohen's d values.

	Mean change	t =	p =	*Cohen’s d*
**FFMQ overall**	12.6	3.2	0.007	0.59
**FFMQ observing subscale**	2.54	2.37	0.036	0.45
**FFMQ describing subscale**	2.38	2.28	0.042	0.41
**FFMQ non-reacting subscale**	2.61	2.71	0.019	0.69

### Qualitative analysis

The responses for each of the two questions were initially analysed separately, with a range of sub-themes identified. Positive effects in participants’ personal life and clinical practice were noted.

### Personal benefits

A common response amongst participants was the change in their relationship with their emotions, particularly in their capacity to identify, observe, and subsequently regulate them, as evidenced by the following examples: “Better able to notice emotions”, “Increased emotional regulation”, “Checking in more often with feelings and sensations”, “making room and allowing”, “awareness of what I am experiencing in the moment”, “I am less emotionally reactive”, “I am more aware of my emotions and in better control of them”.

Tolerance of uncertainty was another common benefit that arose. Some participants indicated that the intervention led to a decrease in their intolerance of uncertainty, a psychological phenomenon that is implicated in anxiety and related to one’s ability to productively and functionally appraise unknown situations. Responses included: “Helped with decreasing attachment to things I can’t control”, “Tend to have a greater sense of agency with challenging life situations”, “… using STOP and RAIN to pause, step back, identify, make space has definitely help me to cope better with uncertainty”. Many participants commented on the benefits of the practical elements of the workshop and the subsequent increase in their therapeutic skillset, including the STOP and RAIN mnemonics (tools to remember how to navigate stressful situations).

Participants of this study indicated that having a dedicated space for self-reflection, particularly among a safe and supportive group, encouraged them to engage in more self-love which related to a theme of self-compassion. Responses included: “Given myself permission for self-care … ”, “More loving kindness to myself …”, “Being more loving/accepting of myself”.

Some participants seemed able to translate the increase in self-care into motivation to improve their regular routine. Responses included: “It has rekindled my interest in mindful practice. Not only doing the homework but doing reading”, “It has brought me back to regular practice which I’m thankful for”, “Awareness of the importance of committing to a regular daily practice.”, “Accountability for practice = more structured practice”, “Practice self-compassion more frequently”.

Responses included: “Using “STOP’ fairly often”, “Gave me ‘tool’ to use with my child. Re-affirmed some positive skills I use/have already with ‘data’ (i.e., name emotion, loving kindness, touch.)“, “Usefulness of STOP and RAIN – practical techniques … using STOP and RAIN to pause, step back, identify, make space has definitely help me to cope better …”, “Gave me skills for relaxation when I am stressed.”, “I particularly enjoyed the “what brings you joy” exercise and found myself asking my loved ones that question”.

Finally, participants frequently discussed the positive impact that the above factors had on their interpersonal relationships: Responses included: “It has improved my relationships with my friends and family. I am less emotionally reactive with my spouse and children”, “I’m more present in my relationships”, “… more loving … of others”.

### Clinical practice

Many participants indicated that they believe they became more effective in their interactions with clients, through a change in their capacity to manage their own emotions and distress tolerance, indicating an increase in mindfulness when working with patients: Responses included: “Easier to ‘stay’ with difficult patient and situations”, “I find myself more present with clients and less reactionary … to what clients are saying and feeling”, “I’m more aware of my ego slipping into the room and the habit of thinking of responses to clients before they are even finishing talking”, “Listening more and being quiet more”.

As well as through changes in their capacity to cope with workload and related stressors; responses relating to this theme included: “More loving kindness to myself as expressed by offering myself more time between sessions …”, “Learned skills to relax when stressed and this has enabled me to do more i.e., to be more efficient in finishing my tasks”, “Helped with work productivity and ability to slow down and cope with workload more effectively”, “I have a tendency to jump to problem solving/change strategies when not mindful – using STOP has helped me STOP, validate, then work toward change”.

Responses also indicated that participants are likely to utilise the knowledge from the intervention when providing psychoeducation to clients: “Using the breath to help with present moment connection to my clients”, “I loved reflecting daily on some of the slogans … using these within my work”, “Helping clients connect more to their breath/sensations in their body”, “encouraging it more with patients”, “Realisation of the importance of sharing this practice with my client”, “I have shared the STOP technique with pain clients and children and their families and they inform of finding it helpful.”

Lastly, participants report a noteworthy change to the general relationship they have to their workplace as well as improvement to their workplace relationships: Responses relating to this theme included: “Less manifested irritation at other co-workers”, “It has provided an opportunity to engage with colleagues in a different way and a greater sense of community (in a small way) which could grow …”, “I have been taking more initiative at work … I feel more connected to my colleagues”

### Qualitative synthesis

Overall, the major themes of this study, personal and clinical benefits of mindfulness, can be conceptualised in the context of improvement in two key interpersonal domains. The first is emotional reactivity. Several participants reported a marked reduction in emotional reactivity, which tended to be described as a sense of being able to better notice their own emotional reactions and reflect upon them without immediately responding. This ability to “stop and reflect” was frequently associated with the respondent’s perceived improvement in their ability to listen and communicate. The second is listening and communication. Several participants described that they felt able to listen more deeply to clients during clinical work when emotionally distressing topics were being explored. Finally, a large proportion of participants described that they were able to communicate more effectively with family members and professional colleagues.

Several participants discussed how the reduction in emotional reactivity allowed them more time to reflect on responses, rather than reacting to the emotion of the situation. Subsequent changes in listening and communication were often directly related to the reduced emotional reactivity. As such, it appears that when clinicians are able to advance their mastery over their own emotional processes, they are able to more effectively attend to other’s needs. This process is supported by the commonly used models of transference/counter-transference as well as mindful practice theory. ^44^^,^^45^ Likewise, the combination of the above factors seems to have a commonly shared improvement on personal wellbeing through self-care, self-love, and increased connectedness to others.

## Discussion

This study showed that a short, validated mindfulness course enhances components of, and overall mindfulness in mental-health clinicians, including psychiatrists. Critically, this finding was supported in the participants’ subjective reporting of their experience, which provided more nuanced insight into how the benefits might manifest themselves, primarily through the emotional, communication, and relationship benefits of the program. This study provides a novel synthesis of quantitative and qualitative data relating to the impact of mindfulness training in the sample.

The findings from this study validate the results of previous research, indicating mindfulness is protective against burnout, and provides valuable insight into possible contributing factors. ^46^ Participants reported that after the intervention they were able to listen to clients more deeply and communicate with patients and family more effectively, themes directly related to the *observation and describing* FFMQ subscales. Both *observing and describing* have been positively correlated with active listening and empathy, which in addition to the clinical and therapeutic benefits, are likely to be protective against burnout. ^47^^,^^48^

Non-reactivity contributes to emotional resilience by way of allowing one’s emotions to occur without getting carried away. ^49^ An increase in the non-reacting FFMQ subscale post MAPs was matched by participants’ narrative reports of reduced emotional reactivity. Emotional reactivity is directly correlated with burnout. ^50^ Thus, moderating this with mindfulness techniques provides a direct mechanism of burnout prevention.

There are a number of limitations to this study. Self-selection bias may have selected for those clinicians more ‘psychologically minded’. Inherent limitations did not allow for blinding or control groups. Controlling for confounding variables such as the effects of medication or changes to the participants usual psychological treatment or social circumstances may have impacted the results. Furthermore, while the small sample size sufficed for the purpose of the pilot program, it will have almost certainly impacted on the statistical significance. High pre-test mean scores could have resulted in a ceiling effect, including awareness. Whilst no change in the mean PSS scores was observed, this could be explained by increased clinical demands and stressors in October, November, and December which is typically a busy time of the year for professionals in this field. Further studies can explore this through implementing numerous trials across the year.

Despite the limitations, this study suggests personal and occupational benefits of mindfulness training in mental-health workers through enhanced resilience and ultimately prevention of burnout in mental health clinicians. To the best of the authors’ knowledge, this is the first mixed-methods examination of a mindfulness intervention in the sample population. The subject would benefit from further research using a larger sample size, which would also provide valuable information to support the scalability and practicality of such an intervention.

## Data availability

### Underlying data

Figshare: 2016 Mindfulness Stats Complete.xlsx. https://doi.org/10.6084/m9.figshare.16566135.v1. ^51^

This project contains the following underlying data:

- Amherst 2016 Mindfulness Stats Complete.xlsx: Participant demographic information
- PrePostPostRData_FFMQxlsx.xlsx: Pre- and post-test Five Facet Mindfulness Questionnaire scores
- PrePostRawDataPSS.xlsx: Pre- and post-test Perceived Stress Scale scores

Figshare: De-identified qualitative responses. https://doi.org/10.6084/m9.figshare.16757029.v1. ^52^

This project contains the following underlying data:

- Qual responses.pdf: Written responses to the two qualitative questions

Data are available under the terms of the Creative Commons Attribution 4.0 International license (CC-BY 4.0).
