# Supplementary material for: The benefits of mindfulness in mental healthcare professionals
Source: F1000Res. 2022 Jan 31;10:1085. Originally published 2021 Oct 25. [Version 2] doi: 10.12688/f1000research.73729.2 (PMC8943343; doi:10.12688/f1000research.73729.2)
Supplement: Peer review history

# Peer review: Reviewer response for version 2

**DOI:** 10.5256/f1000research.120773.r121726

**Published:** 2022-03-23

**Authors:** Veena S. Singaram

**Affiliations:** 1 School of Clinical Medicine, College of Health Sciences, University of KwaZulu-Natal, Durban, South Africa

Revisions are accepted. Submission is accepted for indexing.

Is the work clearly and accurately presented and does it cite the current literature?

Partly

If applicable, is the statistical analysis and its interpretation appropriate?

I cannot comment. A qualified statistician is required.

Are all the source data underlying the results available to ensure full reproducibility?

Yes

Is the study design appropriate and is the work technically sound?

Yes

Are the conclusions drawn adequately supported by the results?

Partly

Are sufficient details of methods and analysis provided to allow replication by others?

Partly

Reviewer Expertise:

Health Professions Education

I confirm that I have read this submission and believe that I have an appropriate level of expertise to confirm that it is of an acceptable scientific standard.

# Peer review: Reviewer response for version 1

**DOI:** 10.5256/f1000research.77400.r101856

**Published:** 2022-01-18

**Authors:** Veena S. Singaram

**Affiliations:** 1 School of Clinical Medicine, College of Health Sciences, University of KwaZulu-Natal, Durban, South Africa

The authors pilot a mindfulness program (MAPs) with a group of health practitioners, making this study a highly topical and useful study since this group of practitioners (and others) has been found to undergo symptoms of burnout in healthcare settings. The authors found that the MAPs program had positive benefits for their participants, both personally and professionally.

 The study may be outdated due to the outdated references and the absence of current findings. Recent findings on mindfulness and healthcare professional wellbeing literature have proliferated the field of healthcare professional wellbeing especially since the advent of COVID-19, causing the authors to miss out on current developments in the field of healthcare worker wellbeing and burnout. This absence is understandable since the data were collected before COVID-19 began. Perhaps a word or two from the authors to signal and explain what may appear anachronistic to some readers.

 The authors also comment on the participants’ high pre-test mean scores on the FFMQ and one wonders whether this may have been due to participants’ previous experiences with mindfulness-based programs and whether this does not partly explain the ceiling effect the authors speak about in their limitations. 

 Though the study design was apt, the literature review and discussion spoke of burnout, yet the assessment tested perceived stress scales (PSS). Burnout is not the same as stress, thus I would suggest explaining why PSS was used instead of a burnout inventory assessing the different components as described in the first line of the literature review. Or, I would suggest changing the literature review and conclusions commenting on stress levels instead of burnout.   

 In terms of details of qualitative analysis, the steps were not described enough to make it replicable. Please add a description of how exactly the data was collated and analysed. 

 Otherwise, the study is well put together, well written, and brings important attention to a group of healthcare practitioners professionally mandated to the mental well-being of others.

Is the work clearly and accurately presented and does it cite the current literature?

Partly

If applicable, is the statistical analysis and its interpretation appropriate?

I cannot comment. A qualified statistician is required.

Are all the source data underlying the results available to ensure full reproducibility?

Yes

Is the study design appropriate and is the work technically sound?

Yes

Are the conclusions drawn adequately supported by the results?

Partly

Are sufficient details of methods and analysis provided to allow replication by others?

Partly

Reviewer Expertise:

Health Professions Education

I confirm that I have read this submission and believe that I have an appropriate level of expertise to confirm that it is of an acceptable scientific standard, however I have significant reservations, as outlined above.

# Response

**Published:** 2022-01-28

**Authors:** Ashwin Varghese

**Affiliations:** Monash Medical Centre, Australia

We would like to thank the reviewers for their valuable input. We have addressed each comment in the revised submission. Specifically, we have expanded on the correlation between perceived stress and burnout, provided evidence to support this relationship, and explained why the PSS was used. We have provided a more thorough explanation of data collection and analysis. We have addressed the potential impacts of COVID-19 on the findings and made comments on the possible limitation of not knowing participants' previous experience with mindfulness.

# Peer review: Reviewer response for version 1

**DOI:** 10.5256/f1000research.77400.r100017

**Published:** 2021-11-22

**Authors:** Dr. Richard Mottershead

**Affiliations:** 1 Ras Al Khaimah College of Nursing, RAK Medical and Health Sciences University, Ras Al Khaimah, United Arab Emirates

Thank you for allowing me the opportunity to review this research article paper. I have read the article with interest and have the following comments to make:

 This is a well-composed paper, which will be of interest to all healthcare practitioners working within health and social care settings. The authors are embedded within the practice and seek to make long-standing contributions to the research literature and clinical practice for health and social care professionals through the advent of mindfulness techniques which consequently improve clinical effectiveness. The paper proposes a holistic benefit of enhanced mindfulness from engagement with service users to family members. The necessity for health and social care works need to be within the moment, not only within work but in family life which necessitates positive mental health and well-being in all aspects of life.

 It is an approach to care that advocates self-love and self-care which are often secondary outcomes for health and social care professionals. The authors identify the challenges of generalising results and snowballing techniques that may lead to attracting participants willing and able to engage and adopt highlighted practices but notwithstanding. The research is an engaging insight to adopting evidence-based practices to improve service delivery as well as staff mental well-being. 

 The paper will undoubtedly be followed by others and I wish the authors well. Their work seeks to establish long-term benefits to the health and well-being of practitioners that deserve support in maintaining and enhancing resilience which will of course benefit service user care and recovery.

Is the work clearly and accurately presented and does it cite the current literature?

Yes

If applicable, is the statistical analysis and its interpretation appropriate?

Yes

Are all the source data underlying the results available to ensure full reproducibility?

Yes

Is the study design appropriate and is the work technically sound?

Yes

Are the conclusions drawn adequately supported by the results?

Yes

Are sufficient details of methods and analysis provided to allow replication by others?

Yes

Reviewer Expertise:

I am a qualified researcher, mental health practitioner with experience of supporting health and social care practitioners mindfulness and mental well-being.

I confirm that I have read this submission and believe that I have an appropriate level of expertise to confirm that it is of an acceptable scientific standard.
